# Validating the diagnostic accuracy of medical certification: a population-level comparison between verbal autopsy and Saudi medical records causes of death of deceased with type 2 diabetes

**DOI:** 10.1080/16549716.2024.2448382

**Published:** 2025-01-20

**Authors:** Faleh Alyazidi, Deler Shakely, Fawaz R. Alyazidi, Max Petzold, Laith Hussain-Alkhateeb

**Affiliations:** a School of Public Health and Community Medicine, Institute of Medicine, Sahlgrenska Academy, University of Gothenburg, Gothenburg, Sweden; b Department of Public Health, College of Health Sciences at Al-Leith, Umm Al-Qura University, Al-Leith, Kingdom of Saudi Arabia; c Infectious Diseases Control Department, Executive Directorate of Preventive Medicine, Makkah Healthcare Cluster, Makkah, Kingdom of Saudi Arabia

**Keywords:** Causes of death, medical certification, health system, verbal autopsy, Saudi Arabia, mortality statistics, health policy

## Abstract

**Background:**

In contexts where certifying causes of death (COD) is inadequate – either in industrialized or non-industrialized countries – verbal autopsy (VA) serves as a practical method for determining probable COD, helping to address gaps in vital data.

**Objective:**

This study aimed to validate the diagnostic accuracy of medical certifications at a population level by comparing COD obtained from medical records against those derived from VA in Saudi Arabia.

**Method:**

Death records from 2018 to 2021 were collected from a type 2 diabetes mellitus register at a major specialist hospital in Makkah. Three hundred and two VA interviews were completed with deceased patients’ relatives, and the probable COD was determined using InterVA-5 software. Lin’s concordance correlation coefficient was applied to examine similarities of the cause-specific mortality fractions (CSMFs) based on International Classification of Diseases chapters from both verbal autopsy causes of death (VACOD) and the physician review causes of death (PRCOD).

**Results:**

Overall, the findings demonstrated a moderate level of concordance of COD at the population between VACOD and PRCOD. However, the CSMFs for various COD categories derived from both sources showed a broad spectrum of absolute differences, with the largest disparities observed among the most prevalent COD categories.

**Conclusion:**

PRCOD was found to overestimate population-level endocrine/metabolic and respiratory disease COD while underestimating circulatory disease, demonstrating medical certification challenges. Conversely, affirming previous literature on prevalent COD in Saudi Arabia, VA appears to deliver a plausible assessment, further strengthening its potential to integrate within the Saudi health system towards an augmented medical certification process.

## Paper context

- **Main findings:** There is a moderate concordance between the reference standard verbal autopsy causes of death and the physician review causes of death at the population level, with apparent discrepancies among the most prevalent causes.
- **Added knowledge:** Verbal autopsy is a useful standardized tool for medical certification in Saudi Arabia.
- **Global health impact for policy and action:** Verbal autopsy data can be incorporated into global health statistics to monitor and address key public health challenges related to major causes of death.

## Background

Ascertaining medical cause of death (COD) continues to pose major challenges in both industrialized and non-industrialized countries [1,2]. Approximately half of the global mortalities pass unrecorded, particularly in low- and middle-income countries, as civil registration and vital statistics (CRVS) systems are mostly deficient [3,4]. This critical deficiency is exacerbated by the quality of medical certification worldwide, which is often inconsistent and of suboptimal standards, being typically either incomplete or unreliable [5]. As stressed by the World Health Organization (WHO), numerous countries will continue to fail to institute functional CRVS systems, which may impact the quality of medical certification data and pose obstacles to utilizing timely and meaningful information [6,7].

Data on the distribution of COD within countries is often generated from the CRVS, where declarations of death are supposed to be certified by physicians based on the International Classification of Diseases (ICD) [8,9]. In 2010, approximately 24% of deaths in South Africa were of suboptimal COD information, mainly due to inaccurate completion of death certificates by doctors, despite extensive health system re-engineering and improvement in South Africa [10]. Similarly, a multihospital-based study of high inpatient mortality rates in the United States found that approximately 45.8% of death certificates were completed incorrectly [11]. The study revealed a tendency to overreport heart disease and renal disease, while cancer was often underreported as an underlying COD.

Diabetes mellitus is a major public health challenge worldwide, and Saudi Arabia is no exception, with increasing prevalence rates of this chronic condition [12,13]. Unlike other illnesses that have a significant burden in developing countries, non-communicable diseases (NCDs) are seen as a challenge to low-, middle-, and high-income countries alike [14]. In Saudi Arabia, NCDs burden is prevailing [15], particularly with regard to type 2 diabetes mellitus (T2DM), which has an estimated prevalence of approximately 17.7% [16]. T2DM is a chronic illness marked by a high level of complexity and often presented as co-morbid, which may complicate the COD ascertainment by medical opinion.

Although Saudi Arabia is classified as a high-income country [17], the annually published mortality statistics lack sufficient detailed information on the burden of NCDs [13]. Previous studies in Saudi Arabia have highlighted gaps in the causes of mortality data and challenges that result in unreliable assignment of ICD codes to medical certifications [18–20]. For instance, death reporting faces challenges due to varying death certification practices across different health service sectors and the absence of a standardized electronic reporting system [18,21]. This inconsistency raises concerns regarding the reliability and completeness of CRVS in the region, as differences in the forms used in certification cause misclassifications [21]. Despite the Saudi healthcare system’s efforts to improve the quality and availability of mortality data and to ensure complete and reliable medical COD following the ICD coding system, the current state remains unsatisfactory [20], with unpublished reports based on expert opinions suggesting that this process is likely facing technical, operational, and cultural challenges.

Furthermore, previous literature has identified a deficiency in the context of death certification accuracy, as evidenced by findings from a published report that highlighted inaccuracies in death certification at two referral hospitals in Riyadh City [21]. The report concluded that there is a need to improve accuracy by implementing rigorous measures, such as training in ascertaining COD, and using a unified system of death certification for all hospitals. Although the literature on Saudi CRVS and the estimated gaps in COD information is limited, a recent national-level study of over 29,291 deaths revealed that about 14% of deaths were of ‘ill-defined’ causes and 5% as ‘null’ causes [20]. These deficiencies highlight diagnostic issues associated with medical certifications, particularly where medical autopsies are limited to homicide or suicide-suspected occasions [21].

In contexts where medical death certification is limited or unavailable, the verbal autopsy (VA) method can be a feasible alternative to fill existing gaps by scanning the death profile at a population level [22–24]. The fundamental concept underlying VA involves conducting a standardized interview with close caregivers or family members most knowledgeable about the deceased circumstances, signs, and symptoms leading to death; the resultant data obtained from the interview are subsequently processed to determine probable medical COD [4,22,23]. Although physicians traditionally process VA information to determine a likely medical COD, automated software tools like InterVA-5 have been validated in various settings, and are increasingly used in assigned COD as they offer a faster, cheaper, and consistent approach [4,25–27].

VA facilitates a validated process to determine COD, and the data derived from VA are increasingly applied for health planning, priority setting, and research [28]. Furthermore, VA is a standardized instrument extensively validated in various contexts and by categories of COD, and it is considered a reliable source for addressing gaps in ascertaining COD [25,29]. It is recommended that all countries, especially those with incomplete and inadequate COD statistics, conduct studies to validate and review the quality of their hospital COD data [9]. Autopsies are the gold standard for analyzing medical conditions and COD, but they often face challenges in practical application due to logistical, cultural, or economic reasons associated with this process [30,31]. Nevertheless, this highlights the need for a systematic and practical alternative tool that can effectively complement medical death certification and serve as a reliable reference for comparison.

VA is routinely conducted across registered and unregistered populations, employing standardized WHO VA questionnaires to systematically capture information on COD through structured and narrative sections. However, in the realm of Saudi health policy research, studies aimed at examining the diagnostic accuracy of medical certifications using a validated and standardized tool, such as the VA, are lacking. Therefore, this study aims to validate the diagnostic accuracy of medical certifications at a population level, explicitly comparing the COD reported within medical records of the deceased from T2DM with those COD obtained from the VA tool, which will serve as a presumed reference standard.

## Methods

### Study setting

This is a sub-national register-based cross-sectional study. The study included all deaths identified in the T2DM register of Alnoor Specialist Hospital in Makkah City, Saudi Arabia. Alnoor Specialist Hospital is a tertiary hospital with a capacity of 500-bed and it provides tertiary healthcare services to all residents and visitors of the Makkah region [32]. Alnoor Specialist Hospital is a key public hospital serving a population of 2.5 million, making a significant contribution to healthcare services in the region. The hospital also serves a large proportion of individuals with T2DM, as it has a specialized Diabetic and Endocrine Centre [33], where many patients with this condition follow up and receive ongoing care.

In Saudi Arabia, death certification is typically performed by two physicians without necessarily stating their qualifications or positions [19]. Although Saudi literature on the process of medical certifications is limited, a published study assessing death certification accuracy at two referral hospitals in Riyadh indicated that the death certification procedure involves numerous steps, which can differ between hospitals [21]. At King Khalid University Hospital in Riyadh, the responsible physician (e.g. consultant or resident) completes a death report in both Arabic and English, which is then signed by two other physicians, at least one of whom must be a consultant. Notably, separate Arabic and English forms are utilized, and the information is handwritten. While the English report is retained in the file, the Arabic form is sent to the mortuary. A copy of the Arabic death report is submitted to the Office of Mortuaries and Births, an administrative branch of the Ministry of Interior, by the relatives of the deceased, whereupon a death certificate is issued [21].

### Study sample and sampling process

Data from 1906 death records between 2018 and 2021 were collected from the T2DM medical register at the studied hospital. These records included essential details such as the date and place of death, sex, age, nationality, and contact information of their next of kin. A sample size of 311 deceased individuals was computed from a finite population (sampling frame of 1609 diabetes mellitus-related deaths) using a confidence level of 95%. A 50% cut-off of the population estimated proportion was used in the sample size formula as the most suited parameter when no prior knowledge exists and to minimize any possible underestimation of the sample size [34].

Given that the VA concept is entirely novel to Saudi culture, and VA is often associated with emotional distress such as grief, guilt, helplessness, frustration, anger, and anxiety [30], a response rate of about 50% was assumed. To achieve the pre-estimated sample size of 311 for this study, 700 cases were selected using simple random sampling from the overall 1609 deaths of the T2DM register. Their relatives or close caregivers were then contacted for the VA interviews. Out of these, only 302 VA interviews were successfully completed and retained, after excluding cases where potential participants refused to take part, or where contact information was incorrect or unreachable.

### Data collection and management

Death records retrieved from the medical register included ICD codes indicating the main COD and the preliminary diagnoses representing initial assessments of COD at the hospital. Additionally, contemporaneous with the VA interviews, we noted socio-demographic information disclosed by interviewees, including details such as the deceased’s marital status and educational background. The educational levels were stratified into distinct categories: illiterate, basic education (encompassing primary, secondary, and high school), and advanced education (comprising college and university-level education). The VA respondent’s relationship with the deceased was categorized into two groups: first- and second-order relationships, as described in Table 1.

**Table 1.** Respondents’ relationships to the deceased.

First-order relationships	Second-order relationships
Son	Nephew
Brother	Uncle
Wife	Cousin
Husband	Friend
Sister	Employer
Daughter	
Grandson	
Stepson	
Son-in-law	

Binary classification was used to indicate the marital status of the deceased, with categories of ‘single’ (including divorced and widowed) and ‘married’. The place of death was also recorded and processed as binary data, specifically denoting whether the death was an in- or out-of-hospital event. The COD ascertained from the VA interviews and those reported by the medical service were subsequently classified into broader categories, following the ICD-10-chapter level.

### VA procedures

The VA interviews were conducted with the relatives or close caregivers of the deceased who died between 2018 and 2021, using WHO VA 2016 questionnaire [35]. The COVID-19 pandemic restrictions at the time of fieldwork in Saudi Arabia precluded the possibility of in-person VA interviews, necessitating the exploration of alternative methods. Consequently, telephone interviews were sought as a viable substitute for conventional face-to-face interviews, each lasting an average of approximately 21 minutes. The corresponding author, assisted by a trained research assistant, conducted VA interviews. The families were contacted for scheduling suitable time-slots for conducting the VA interviews.

A comprehensive explanation of the VA interviews and their questions was provided, with emphasis that respondents should seek further clarifications if any question was unclear, and video calls were used whenever needed for enhanced communication. Prior to the VA initiation, 10 VA interviews via phone were piloted to refine the process. Interviews were carried out exclusively with individuals who speak Arabic or English. While this approach helps maintain consistency and accuracy of VA data and mitigate potential language barriers, it may reduce the generalizability of the study’s conclusions to the highly heterogeneous Saudi population in Makkah City. Overall efforts were undertaken to address the challenges presented by the remote communication method while ensuring the study’s outcomes maintain a high level of quality. These challenges included technical issues such as poor signal quality and network instability, which led to an overall prolonged VA interview process.

The data collection process employed the Open Data Kit (ODK) application on Android smartphones, wherein the interviewers gathered all the information. The VA data inputs were thoroughly reviewed and securely stored in the research institute’s electronic databases, after the complete removal of any personal identifiers. The VA data were subsequently entered into a Microsoft Access database, with the participants’ study IDs serving as the prime key for merging the VA data with the background and characteristic information. To ensure confidentiality and anonymity, any identifying information about the deceased or their families was removed post-interview. Subsequently, the original data were replaced with a newly encrypted dataset that had anonymized IDs for analysis and storage, thus the researchers were blinded to participants’ identities (participants were informed of this in the participant information sheet, and prior to commencing interviews).

### Data processing and analysis

Each VA interview’s data were processed using the InterVA-5 software, which assigns up to three likely COD per WHO VA cause categories. InterVA-5 uses a Bayesian probability model to determine the most probable COD and their corresponding likelihoods for each VA case [23,29,36]. The InterVA-5 model uses Bayesian probability, which combines prior knowledge (e.g. medical experts’ opinion) with relevant available data (e.g. data gathered from VA), which can collectively augment the model outputs [26,37]. This methodological process used in the InterVA-5 model was explained in full by Byass et al. [38].

The COD from medical records was presented as a single COD, whereas InterVA-5 determined up to three probable causes for VA data, aligning with the ICD-10 codes. Generated COD likelihoods were utilized to calculate population-level cause-specific mortality fractions (CSMFs) for each COD group between physician review causes of death (PRCOD) and verbal autopsy causes of death (VACOD) – the latter was used as a reference standard – and their proportional absolute differences and 95% confidence interval (CI) were calculated. The CSMFs correspond to the proportion of each COD category in relation to the total number of deaths [26,36,37]. The concordance for the CSMFs from PRCOD and VACOD was also calculated using Lin’s concordance correlation coefficient (CCC), with 95% CI at the population level [39]. Based on Pearson’s correlation, the CCC compares the viewpoints between the two measures (as with other correlation tests, CCC has a range of −1 to 1, with 1 representing perfect concordance) [40].

In this study, our main analysis focused on assessing the concordance of CSMFs between PRCOD and VACOD at the population level. Medical CODs in hospitals are often determined by multiple raters (doctors), which complicates individual-level agreement analysis. Therefore, by concentrating on population-level concordance, we aim to apply a more adequate and generalizable assessment approach to assess the accuracy of the mortality data, which is the ultimate goal of public health budget planning and interventional programs.

An additional analysis was conducted to assess the overall agreement between VACOD and PRCOD, which employed the main PRCOD against the three likely VACODs – agreement was declared if the prime PRCOD matched any of the three likely VACOD. Furthermore, a secondary analysis was undertaken to assess the agreement between VACOD and both the main COD and the preliminary diagnoses of COD in medical records – the agreement is established if any of the three likely VACOD matched any of the two PRCODs. All analyses were performed using the statistical package STATA Release 17.0 software.

### Ethics and consent

The research protocol for this study was approved by the Institutional Review Board (IRB) at Makkah Health Affairs, Ministry of Health, Saudi Arabia, under IRB Number H-02-K-076-0321-478. Data collection, analysis, and storage adhered to the ethical guidelines and regulations specified by the IRB and the General Data Protection Regulation, as per the Declaration of Helsinki. Given the nature of the VA process, interviews were conducted remotely via phone, primarily to comply with COVID-19 safety protocols. Additionally, conducting telephone interviews tended to reduce emotional distress, as participants were able to engage in the interview from the comfort zone of their own environment without the potential stress of being in a formal face-to-face setting. This approach is likely able to alleviate feelings of vulnerability or anxiety that some participants might experience during in-person interviews.

As a fundamental component of the VA training, ethical considerations and sensitivity training were emphasized to ensure that bereaved families are approached with care and respect. The interviewers underwent comprehensive training before beginning the VA data collection process. This included comprehensive sessions with VA experts and online workshops that emphasized the objectives and importance of VA interviews, ethical considerations, and step-by-step instructions on conducting the interviews using the standardized WHO VA 2016 questionnaire. Participants were first contacted to schedule most suited time for conducting the interview and were informed of their freedom to pause or discontinue the interview at any point during the process. Furthermore, interviewers were trained how to remain attentive to participants’ emotional cues, offering to pause or change the topic when appropriate and to ensure that the emotional well-being of participants was always prioritized.

All participants were informed in full of the nature and scope of the study, and the applicable data gathering, storage, and handling considerations, including the guarantee of absolute anonymity, as well as their right to refuse to participate or to withdraw from the study at any time prior to their data being automatically rendered anonymous. Those who voluntarily agreed to participate were requested to provide formal verbal consent, which included the option to decline or withdraw. Informed consent was documented through the IRB-approved consent form. Upon declaration by the respondent, the VA interviewer recorded the date, time, and state of approval or rejection of the respondent. Rejected interviews were discarded from the dataset and analysis. Given that this is verbal consent, the VA interview assistant (FRA) witnessed the consent procedure.

## Results

During the timeframe from 2018 to 2021, a total of 1609 deaths were identified at Alnoor Specialist Hospital T2DM register. Out of these, 302 participants who were either relatives or caregivers of the deceased were contacted and consented to conduct the VA interview. Only three of the respondents were female. The InterVA-5 software identified either one, two, or three likely VACOD. Table 2 illustrates the background and characteristics of 302 deaths.

**Table 2.** Background and characteristics for 302 deaths in Makkah region, Saudi Arabia.

Background characteristics	Distribution of deaths *n* (%)
**Age group**	
34–59	68 (22.5)
60–69	88 (29.2)
70–79	84 (27.8)
≥80 years	62 (20.5)
**Sex**	
Female	126 (41.7)
Male	176 (58.3)
**Nationality**	
Saudi	248 (82.1)
Non-Saudi	54 (17.9)
**Respondent’s (interviewee) relationship to the deceased**	
First-order relationships	260 (86.1)
Second-order relationships	42 (13.9)
**Education level of the deceased**	
Illiterate	121 (40)
Basic (primary, secondary, high school)	137 (45.4)
Advanced (university)	44 (14.6)
**Year of death**	
2018	28 (9.3)
2019	69 (22.8)
2020	161 (53.3)
2021	44 (14.6)
**Marital status**	
Single	116 (38.4)
Married	186 (61.6)
**Place of death**	
Home	60 (19.9)
Hospital	242 (80.1)

Table 3 illustrates the CSMFs derived from VACOD and PRCOD, along with their absolute differences and associated 95% CI, using the ICD-10-chapter level. Notably, despite apparent differences, Chapter I (‘Diseases of the circulatory system – I00-I99’), including cardiovascular diseases (CVD) and stroke), chapter E (‘Endocrine, nutritional, and metabolic diseases – E00-E90’), and chapter J (‘Diseases of the respiratory system – J00-J99’) were the most prevalent COD in both VA and physician reviews.

**Table 3.** Cause-specific mortality fractions per chapter categories derived from VACOD and PRCOD for 302 deaths, Makkah City, Saudi Arabia.

ICD chapter and description	CSMF of VACOD	CSMF of PRCOD	Absolute difference (95% CI)
I. Chapter IX. Diseases of the circulatory system (I00-I99)	52.37	20.53	−31.84 (−39.08, −24.60)*
E. Chapter IV. Endocrine, nutritional and metabolic diseases (E00-E90)	17.89	40.4	22.50 (15.49, 29.53)*
**Unknown causes of death**	7.73	2.65	−5.08 (−8.59, −1.57)*
C. Chapter II. Neoplasms (C00-D48)	5.28	5.3	0.02 (−3.55, 3.59)
B. Chapter I. Certain infectious and parasitic diseases (A00-B99)	5.34	2.98	−2.36 (−6.25, −0.23)*
J. Chapter X. Diseases of the respiratory system (J00-J99)	5.23	18.5	13.26 (8.22, 18.32)*
N. Chapter XIV. Diseases of the genitourinary system (N00-N99)	2.87	0.66	−2.21 (−4.30, −0.12)*
V. Chapter XX. External causes of morbidity and mortality (V01-Y98)	1.43	0.00	−1.43 (−2.77, −0.09)*
W. Other external causes of accidental injury (W00-X59)	0.98	0.33	−0.65 (−1.94, 0.64)
K. Chapter XI. Diseases of the digestive system (K00-K93)	0.615	2.65	2.04 (0.02, 4.05)*
X. Exposure to smoke, fire and flames (X00-X09)	0.12	0.00	−0.12 (−0.51, 0.27)
R. Chapter XVIII. Symptoms, signs and abnormal clinical and laboratory findings, not elsewhere classified (R00-R99)	0.10	0.00	−0.10 (−0.46, 0.26)
U. Chapter XXII. Codes for special purposes (U00-U85)	0.00	2.98	2.98 (1.05, 4.87)
Y. Chapter XX. External causes of morbidity and mortality. (V01-Y98)	0.00	1.99	1.99 (0.41, 3.57)*
G. Chapter VI. Diseases of the nervous system. (G00-G99)	0.00	0.99	0.99 (−0.13, 2.11)

CSMF of VACOD: Causes Specific Mortality Fraction of Verbal Autopsy Causes of Death at a population level. CSMF of PRCOD: Causes Specific Mortality Fraction of Physician Reviewed Causes of Death. *Significant absolute difference.

**Figure 1.**
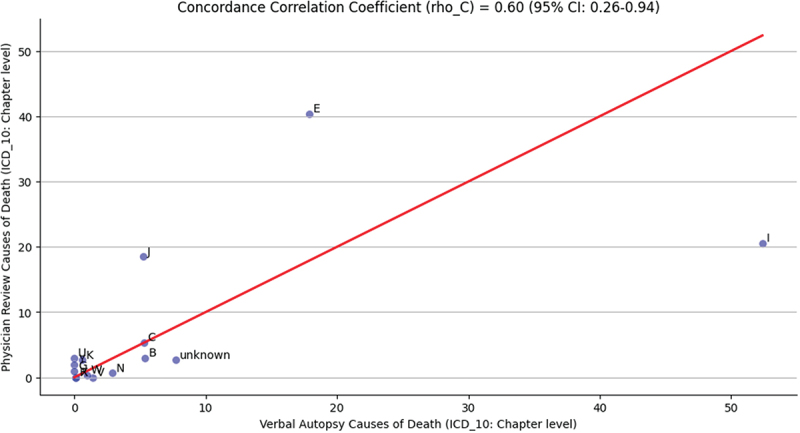
Concordance correlation coefficient analysis for the broader causes of death categories between CSMFs determined by verbal autopsy and physician reviews for 302 deaths in Makkah City, Saudi Arabia.

Figure 1 illustrates the concordance of CSMFs of VACOD and PRCOD at the population level. Based on findings from the CCC, there was a moderate concordance at the population level between (VACOD and PRCOD) in terms of CSMFs of COD categories, with a CCC rho_C = 0.60 (95% CI: 0.26–0.94; *p* = 0.001). The figure highlights apparent disparities, particularly an overestimation of Chapter E (Endocrine, nutritional, and metabolic diseases – E00-E90) and Chapter J (Diseases of the respiratory system – J00-J99) in PRCOD compared to the reference VACOD. Conversely, Chapter I (Diseases of the circulatory system – I00-I99) appears to be underestimated as a COD in physician reviews compared to the reference standard.

Cause of death categories closer to the best-fit line show a higher level of concordance between both sources. Categories falling below the fit line demonstrate an underestimation of the true causes of death, while categories above the fit line indicate an overestimation.

Our findings of the overall agreement between the three likely CODs identified through InterVA-5 software and the prime PRCOD was 28.48% (86 cases). Subsequently, an additional analysis was conducted to further assess the overall agreement, which included comparing the likely COD derived from InterVA-5 with both the main PRCOD documented in medical records and the preliminary diagnoses of COD made at the hospital. This examination led to a noteworthy improvement, with the overall agreement increasing by approximately 37% relative to the initial figure, bringing the revised agreement to 39%, representing 118 cases.

## Discussion

COD data extracted from death certificates were a pivotal metric for evaluating population health, comparing mortality rates nationally and globally [20], and informing discussions on health policy and priority settings [8]. The VA aims to specifically target epidemiological needs at a population level [30], placing importance on accurate estimates of CSMFs for specific populations. Our findings demonstrate a moderate concordance between VACOD and PRCOD at a population level, with a CCC rho_C = 0.60 (95% CI: 0.26–0.93; *p* = 0.001). While apparent disparities exist across the main COD at the population level, both data sources unveil similar patterns, particularly with regard to the three main CODs. In both datasets, circulatory system, endocrine, nutritional, and metabolic diseases, as well as respiratory system diseases, appear to be the main CODs across the Medical Records (75.5%) and VA (79.4%) sources. This consistency across both sources highlights a shared priority for health interventions. It can also indicate common health challenges and facilitate the planning and implementation of coordinated public health strategies, as both data sources point to the same critical health issues.

Although VA can by no means replace the gold standard COD assessment (i.e. the medical autopsy), the VA population-level ascertainment of death categories in this study reaffirms the reliability of VA and what would plausibly be expected in this Saudi population with T2DM. Notably, in alignment with existing literature, the VA-derived COD data in this study appear more plausible compared to those derived from medical records (PRCOD). For instance, based on the VACOD, the leading COD group is attributed to circulatory diseases (52.37%), which certainly falls within the conventionally reported Saudi statistics [20,41,42]. This observation is particularly noteworthy in Saudi Arabia, where CVD is increasingly recognized as a substantial health concern, with an estimated contribution of over 45% to the overall mortality rate [41].

In contrast to the reference standard, it appears that diseases of the circulatory system are being underestimated COD as derived from medical records (PRCOD), whereas diabetes is being disproportionately overreported as a direct COD. This observed discrepancy could be attributed to the common practice of medical certifications, whereby the high prevalence of diabetes in the country could potentially bias the determination of COD [16,43,44]. There is a strong potential for certification bias on the part of physicians when they know that the deceased has T2DM (based on their register information or treatment history), which can likely influence the physician’s medical opinion, compounded by the fact that physicians tend to only assign one COD.

Several factors can influence the accuracy of medical certification of the COD, including the certifiers’ ability to access the deceased medical history and to correctly identify the sequence of events that led to the death, as well as their assessment of the underlying COD [45]. Additionally, there is an interaction between an individual’s age and diseases, where signs and symptoms tend to overlap only to complicate the diagnosis, particularly what we could observe among the elderly group of this study. Moreover, compared to the general population, individuals with diabetes have a two to four-fold higher risk of developing CVD [41]. Hence, more circulatory-related deaths would be expected to appear in this T2DM medical register.

VACOD findings related to endocrinal conditions, mainly represented by diabetes (17.89%), demonstrate greater plausibility than corresponding CSMFs from the PRCOD (40.4%). Diabetes is estimated to contribute to 11.3% of mortality worldwide, with variations observed across regions, ranging from the lowest contribution of 6.8% in the Africa Region to the highest at 16.2% in the Middle East and North Africa [46]. In the Saudi context, diabetes mellitus remains among the top COD, accounting for 13.7 [44]. While diabetes poses a substantial public health concern, and can lead to severe complications, it might be *associated with* COD without necessarily being *the main* COD. From a medical perspective, individuals with T2DM demonstrate a heightened risk of myocardial infarction and sudden death, ranging from two to four times higher compared to those without diabetes, with approximately half of diabetes deaths co-existing with various forms of CVD [47]. This highlights the complexity of causal relationships and emphasizes the need for more nuanced assessments in medical certifications.

The study findings reveal an overestimation of respiratory system diseases by the PRCOD, mainly represented by pneumonia, compared to the VACOD, which is likely influenced by increased reporting during the COVID-19 pandemic (2019–2021). A subgroup analysis of the distribution of respiratory diseases COD by the year of death shows a substantial increase in deaths reported due to this cause category, constituting 75% of all cases in 2020. Despite limited data, this increase likely mirrors the impact of the COVID-19 pandemic on reporting patterns, aligning with expectations in Saudi Arabia and elsewhere, whereby the reported estimation of COVID-19 fatality rate increased from 2% to 3.4% in 2020 [20], warranting a cautious interpretation of medical statistics. The disagreement in the CSMFs of respiratory diseases category between VACOD and PRCOD is consistent with a previously published study in Mexico, where a lower agreement in the CSMFs for causes of chronic obstructive pulmonary disease (COPD) and other infectious diseases was also reported [48]. Nevertheless, our findings imply potential challenges with the VA in determining COD related to this death category, and also highlight concerns regarding medical certification overestimation issues related to these conditions.

In an era characterized by a double or triple burden of diseases, such as the backlog of common NCDs like diabetes, CVD, cancer, as well as mental illness coupled with the emerging climate-sensitive infectious diseases, relying exclusively on the direct COD can result in omitting valuable information regarding causes of mortality and their interactions [49]. While VA is not considered a gold standard, the VA probabilistic approach, which yields up to three likely CODs [23,29], can fill-in gaps in vital data and address unappreciated key contributors of CODs and their interactions at the population level [49].

As evidenced by our subgroup analysis, the overall agreement between the two COD sources witnessed a notable relative improvement of 37%, transitioning from 86 (28.48%) to 118 (39%) instances. This enhancement in agreement was particularly pronounced when evaluating the agreement between the likely COD identified through the VA and the main COD reported in medical records, together with the preliminary diagnoses representing initial assessments of COD at the hospital. Therefore, our study reinforces the role of VA as a valuable method with the potential to be integrated into routine medical certification. This can enhance the reliability and completeness of COD data, particularly for deaths that occur outside the health settings or complex causes. Out-of-hospital deaths may be more prone to inaccurate COD coding or misclassification, as the circumstances surrounding the death are often less well documented compared to in-hospital deaths [50], which can lead to underestimation of disease burden and hinder effective public health responses.

As a pragmatic and validated approach for obtaining COD information, mainly in settings where well-functioning CRVS is underdeveloped [28,30], VA seems particularly relevant for re-engineering the medical certifications practices and national statistics in Saudi Arabia. Although VA is not intended to replace the medical certification process in Saudi Arabia, its integration using innovative and automated software tools would strongly align with the Saudi national agenda towards digitalizing health and societies and strengthening vital information platforms. In circumstances of out-of-hospital deaths, the conventional gold standard of traditional autopsy is often challenging to apply in practice, due to logistical and cultural barriers [30,31]. Additionally, relying solely on physician reviews for certifying COD of unattended deaths may be deemed impractical and often unreliable [31]. Similarly, asymptomatic individuals with mild clinical symptoms might face misdiagnosis or receive ill-defined COD assessments [20]. However, further exploration and assessment is required to answer questions concerning the VA’s cultural acceptability, conducting VA interviews, ethical implications, training, and health system capability to implement this approach.

Despite the rigor applied in our study, it is worth mentioning that the agreement between the two sources may be highly prone to bias by chance due to factors that cannot be completely controlled, given the limitations of our data. Consequently, Although the findings suggest implications for public health policymaking, it is crucial to approach our findings with caution. Furthermore, the relatively small sample size can limit the generalizability of the study findings, which we have cautiously considered when discussing and debating the study results.

Nevertheless, the study sample represents the register data from a major specialist hospital in Makkah City, where most T2DM cases are reported. This study employs the VA as the reference standard for evaluating the diagnostic accuracy of medical certification, relying on a series of international validation studies that affirm the VA’s reliability as a source of vital data. Nevertheless, it is crucial to acknowledge that, despite these validations, a level of uncertainty exists. The method’s accuracy of COD ascertainment is highly dependent on the type of death, the quality of the interview, and the procedures used to assign the COD [36,51]. A variety of factors related to the interviewer, the respondent’s background, or both can influence the quality of the interview and thereafter the VA results [35].

Furthermore, COVID-19 physical distancing measures led to the adoption of VA telephone interviews instead of face-to-face interviews for data collection. Nonetheless, the literature indicates that telephone interviews are equally effective as face-to-face interviews [52,53]. One study indicated that VA telephone interview results were consistent with prior research, highlighting that the method is feasible, well-accepted by both caregivers and healthcare workers and can be used as an alternative to face-to-face interviews without affecting data quality [54].

Moreover, the absence of information on the duration and degree of diabetes control may potentially hinder a comprehensive interpretation of results, due to variations in disease management. For example, a higher mortality rate of circulatory diseases may be expected among individuals with T2DM who have had the disease for an extended period, or who have had poor diabetes management. Finally, the data was collected during the pandemic, and no ICD codes were assigned by InterVA-5 for COVID-19 (there was no defined ICD code at the time). Despite these limitations, conducting remote VA interviews on a small sample in a place where VA has never been practiced, can be viewed as a strength from an ethical perspective. This approach can mitigate the risk of undesirable emotional consequences or consequences related to cultural sensitivities.

## Conclusion

In conclusion, the findings of this study reveal a moderate concordance between the reference standard VACOD and PRCOD at a population level, with apparent discrepancies among the most prevalent COD. However, in line with other cross-sectional patterns in Saudi Arabia, the results of VA appear to deliver a plausible assessment of circulatory and diabetes-related COD, the country’s top listed burden. This stresses the usefulness of integrating a standardized tool like VA to augment the medical certification process in Saudi Arabia, thereby contributing to Saudi national mortality statistics.

## Data Availability

The authors confirm that the data supporting the findings of this study are available upon reasonable request from the main author.
